# Treatment of Myocardial Infarction with Gene-modified Mesenchymal Stem Cells in a Small Molecular Hydrogel

**DOI:** 10.1038/s41598-017-15870-z

**Published:** 2017-11-20

**Authors:** Zhiye Wu, Guoqin Chen, Jianwu Zhang, Yongquan Hua, Jinliang Li, Bei Liu, Anqing Huang, Hekai Li, Minsheng Chen, Caiwen Ou

**Affiliations:** 1 Department of Cardiology, Heart Center, Zhujiang Hospital, Southern Medical University, Guangzhou, 510280 China; 2 Guangdong Provincial Biomedical Engineering Technology Research Center for Cardiovascular Disease, Zhujiang Hospital, Southern Medical University, Guangzhou, 510280 China; 3 Cardiovascular Medicine Department of Central Hospital of Panyu District, Guangzhou, 510280 China; 4 Department of Cardiology, Nanfang Hospital, Southern Medical University, Guangzhou, 510515 China; 5 Department of Cardiology, Shanghai general hospital, Shanghai, 200000 China

## Abstract

The effect of transplanted rat mesenchymal stem cells (MSCs) can be reduced by extracellular microenvironment in myocardial infarction (MI). We tested a novel small-molecular hydrogel (SMH) on whether it could provide a scaffold for hepatocyte growth factor (HGF)-modified MSCs and alleviate ventricular remodeling while preserving cardiac function after MI. Overexpression of HGF in MSCs increased Bcl-2 and reduced Bax and caspase-3 levels in response to hypoxia *in vitro*. Immunocytochemistry demonstrated that cardiac troponin (cTnT), desmin and connexin 43 expression were significantly enhanced in the 5-azacytidine (5-aza) with SMH group compared with the 5-aza only group *in vitro* and *in vivo*. Bioluminescent imaging indicated that retention and survival of transplanted cells was highest when MSCs transfected with adenovirus (ad-HGF) were injected with SMH. Heart function and structure improvement were confirmed by echocardiography and histology in the Ad-HGF-SMHs-MSCs group compared to other groups. Our study showed that: HGF alleviated cell apoptosis and promoted MSC growth. SMHs improved stem cell adhesion, survival and myocardial cell differentiation after MSC transplantation. SMHs combined with modified MSCs significantly decreased the scar area and improved cardiac function.

## Introduction

Myocardial infarction (MI) is the irreversible necrosis of myocardial as a result of oxygen deprivation, which in turn is caused by obstruction of the blood supply. Stem cell therapy has the potential to regenerate cardiomyocytes and replace scar tissue with new cardiomyocytes^1–3^. The aim of cell-based interventions is to limit the extent of damage and prevent organ failure in the early stage of post-infarction by altering the myocardial response to injury^4^. Bone marrow (BM) mesenchymal stem cells (MSCs) have the advantages of easy sampling, multi-directional differentiation potential, and low immunogenicity. Thus, MSCs have become a focus in stem cell treatment of MI. Animal and clinical studies have demonstrated safety and efficacy of MSC transplantation after MI. Additionally, 5-azacytidine (5-aza), a DNA demethylating agent, induces expression of cardiac-specific markers, such as α-myosin heavy chain (MHC) and Nkx2.5, and spontaneous beating rhythmic in rat BM-derived MSCs^3,5^. Due to ischemia, hypoxia and oxidative stress following infarction, the ability of transplanted cells to survive is compromised and the cells are unable to effectively differentiate into myocardial cells, which severely reducing the therapeutic effect of stem cell transplantation^6–8^. This is one of the major barriers for cell therapy in the treatment of MI^9^. Therefore, promoting survival and differentiation of transplanted stem cells through modulation of the cellular microenvironment is a promising approach for successful stem cell therapy^10^.

In recent years, stem cells engrafted with injectable hydrogel has shown great potential to repair necrotic myocardium. Studies have demonstrated that biomaterials can be very useful in enhancing retention of transplanted cells^11–14^ by providing spatial support to transplanted cells and enhancing cell survival. For example, alginate hydrogel improved electrical conduction and increased MSC retention in healed MI^15^, which validating the accessibility and effectiveness of a bio-scaffold^15,16^. Small molecular hydrogels (SMHs) are receiving increased attention in stem cell transplantation. SMHs promote cell adhesion^17^ and can serve as a potential nanocarrier for localized and sustained delivery of doxorubicin *in vivo* ^18,19^. Thus far, supramolecular hydrogel has been widely used in limiting damage following the MI, however, our research on the therapeutic effect of SMHs has been pioneering^20,21^.

**Figure 1:**
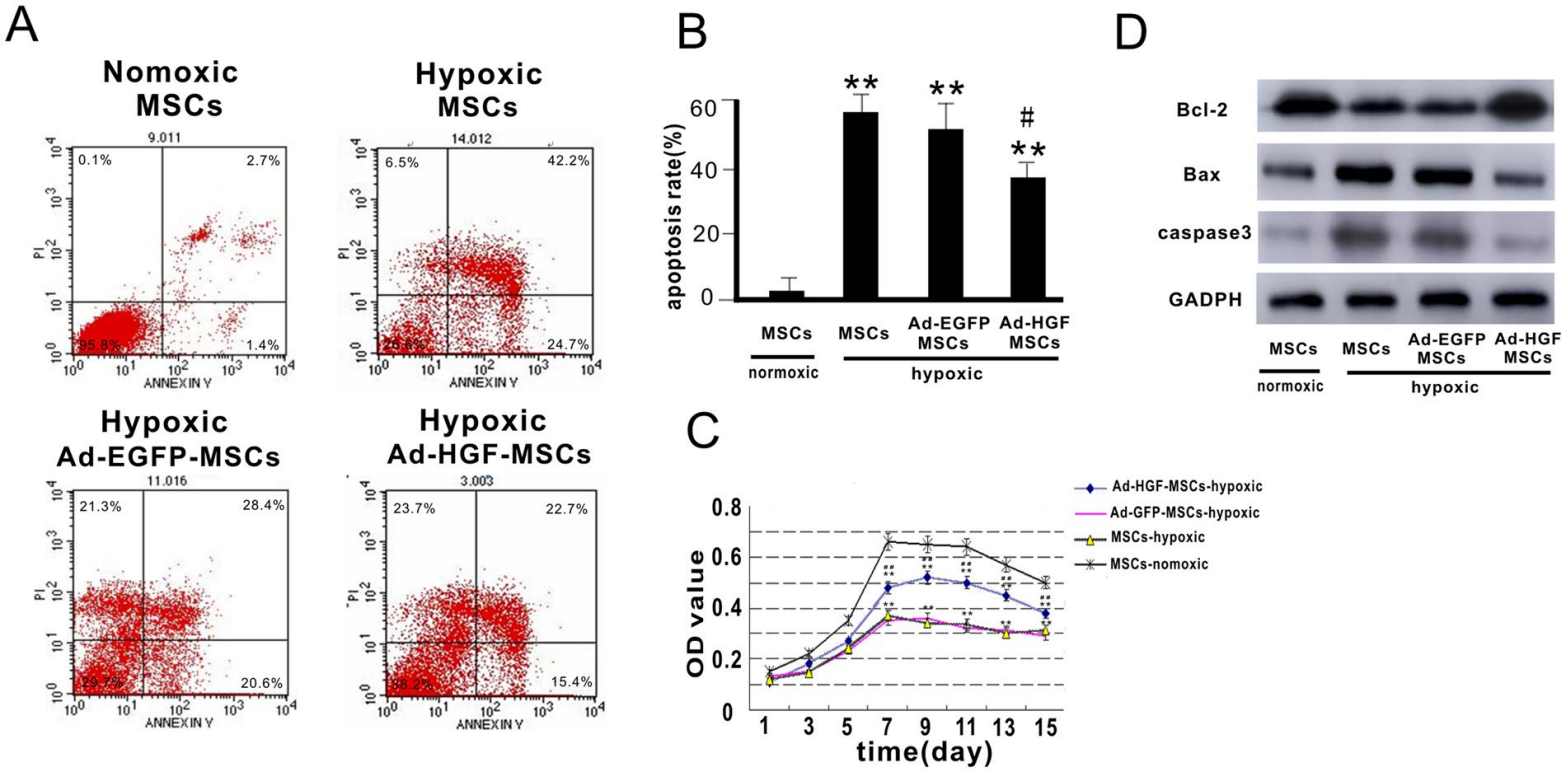
Infection of HGF into MSCs and the effect on MSC growth and apoptosis after. 12 hours of hypoxia. (**A**) Flow cytometry analysis of Annexin V/PI was carried out in MSCs. (**B**) Apoptosis rate was calculated according to Annexin V/PI flow cytometry analysis. (**C**) MTT curve of MSCs infected with Ad-HGF or vector in normoxic or hypoxic conditions. (**D**) Representative Western blot and level of Bcl-2, Bax and caspase-3. Data are mean ± SEM. **P < 0.01 *vs*. MSCs-normoxic, ^*#*^ *P* < 0.5, ^*##*^ *P* < 0.1 *vs*. Ad-EGFP-MSCs-hypoxic. n = 3, One-way ANOVA, then followed by post-hoc Tukey’s test for multiple comparisons.

Hepatocyte growth factor (HGF), a potent agonist for the tyrosine kinase surface receptor c-MET, has antifibrotic, proangiogenic, and cardioprotective effects against stress and injury^16,22,23^. Furthermore, there is evidence of HGF having therapeutic effects after MI in rats^24–27^. Recent research has shown that active microcarriers that continuously release HGF can improve engraftment and repair ability of human adipose-derived stem cells in healing infarcted hearts in rats^28,29^. Here, we report a combined effect using SMHs to carry adenovirus (Ad)-HGF gene-modified MSCs to mitigate MI damage, and discuss the possibility of utilizing a myocardial tissue engineering scaffold material to treat MI (S. Figure 1).

## Results

### The effects of recombinant adenovirus and Ad-HGF on MSCs

The HGF level in MSCs was detected by Western blot (Figures S3). Annexin V/PI flow cytometry analysis was carried out 12 hours after hypoxia or normoxia. The percentage of apoptotic cells was lower in the Ad-HGF-MSCs group (37.83 ± 3.67%) compared to the Ad-EGFP-MSCs (52.87 ± 5.09%) or MSCs (53.07 ± 4.58%) group in hypoxia (*P* < 0.05). We observed a consistent trend in the 3-(4,5-Dimethylthiazol-2-yl)-2,5-diphenyltetrazolium bromide (MTT) assay among the Ad-EGFP-MSCs, MSCs, and Ad-HGF-MSCs groups in hypoxia. Thus, Ad-HGF overexpression remarkably suppressed MSC apoptosis induced by hypoxia (Fig. 1A,B). HGF in the cell supernatant was detected by ELISA (Figure S4). In future research, we will study in depth the mechanism by which HGF mediates apoptosis in MSCs.

MTT analysis showed that MSC growth and proliferation were normal in the normoxic group. However, the optical density (OD) value of the Ad-HGF-MSCs group was significantly increased compared with the Ad-GFP-MSCs and MSCs groups in hypoxia at 15 days (*P* < 0.05). In addition, no statistical difference was observed between the Ad-GFP-MSCs and MSC groups in hypoxia. The results indicate that HGF overexpression had a beneficial effect on cell proliferation under hypoxic conditions (Fig. 1C).

The significant increase in Bcl-2 level was observed in the Ad-HGF-MSCs group compared to the Ad-EGFP-MSCs and MSCs groups 12 hours after hypoxia or normoxia, and there was no significant difference between Ad-HGF-MSCs in hypoxia and normoxic MSCs. In contrast, Bax and caspase-3 levels were markedly increased in the hypoxic Ad-GFP-MSCs and MSCs groups compared to the normoxic MSCs group. We also found no significant difference in Bcl-2, Bax and caspase-3 expression between the Ad-GFP-MSCs and MSCs groups in hypoxia (Fig. 1D).

### Proliferation, survival, and differentiation of MSCs in the presence SMHs

**Figure 2:**
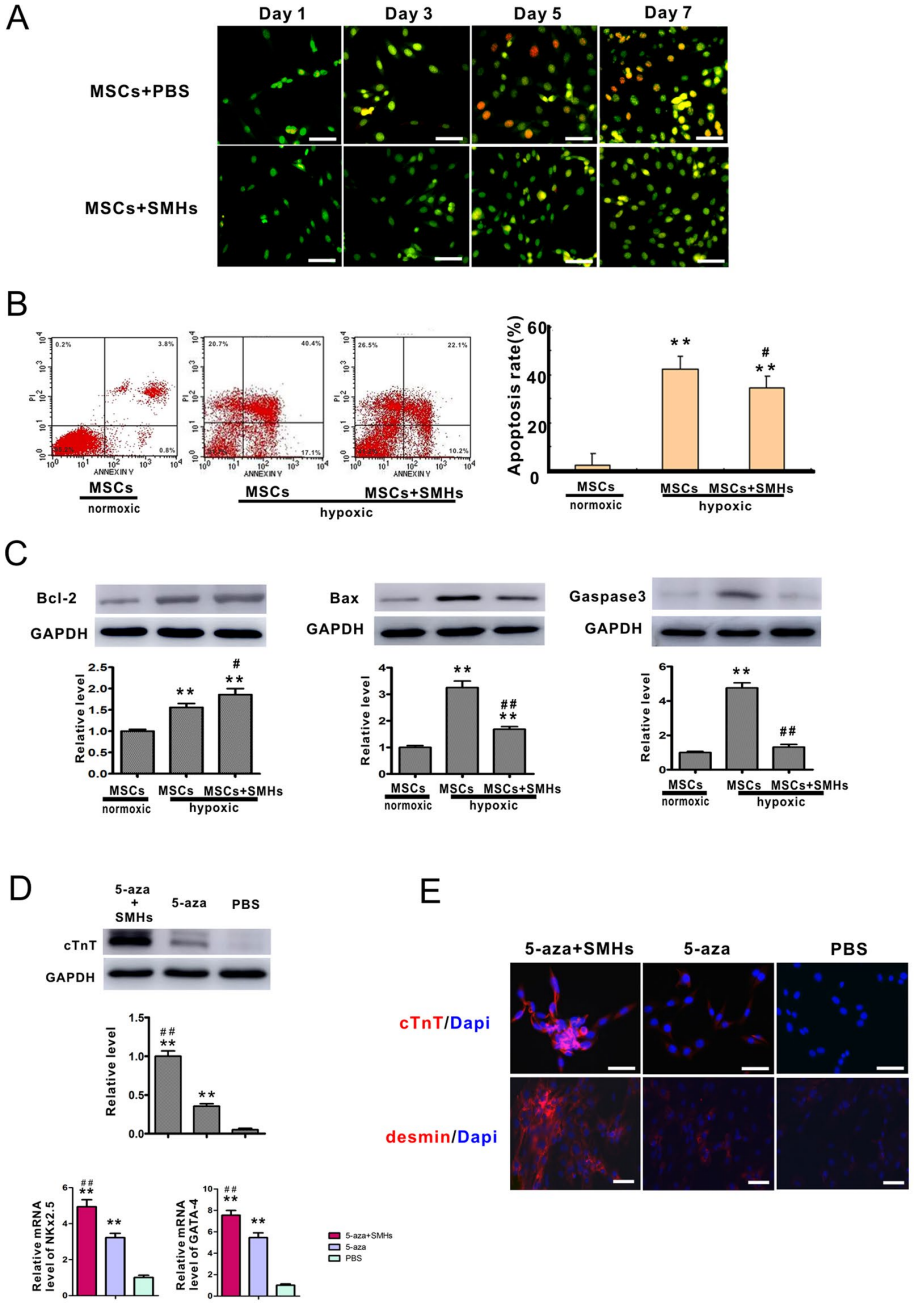
The effect of SMHs on MSC proliferation, survival and differentiation. (**A**) MSCs cultured in SMHs or normal medium were visualized by AO/EB staining, magnification:400x. (**B**) Annexin V/ PI flow cytometry analysis with MSCs cultured with SMHs in hypoxic or normoxic conditions. Data are mean ± SEM. *P < 0.05 *vs*. MSCs-normoxic, ^*#*^ *P* < 0.05 *vs*. MSCs-hypoxic,n = 3. (**C**) Western blot and level of Bcl-2, Bax and caspase-3 in each group. Levels of proteins were quantified by densitometry and normalized against GAPDH, data are mean ± SEM. *P < 0.05 *vs*. MSCs-normoxic, ^*#*^ *P* < 0.05 *vs*. MSCs-hypoxic, all the experiments were repeated three times then selected the representative result for each protein level. (**D**) Representative western blot and the level of cTnT; Level of NKx2.5 and GATA-4 were detected by qPCR. Data are mean ± SEM. **P < 0.05 *vs*. PBS; ^*##*^ *P* < 0.01 *vs*. 5-AZA. n = 3, Levels of proteins and mRNA were quantified by densitometry and normalized against GAPDH, One-way ANOVA, then followed by post-hoc Tukey’s test for multiple comparisons. (**E**) SMHs affected MSC differentiation as shown by red fluorescence in photomicrographs: cTnT, and desmin, Blue: DAPI-stained nuclei, magnification:400x.

Cell apoptosis and necrosis were studied on days 5 and 7 in SMHs *in vitro*. AO/EB staining revealed that the MSCs cultured in SMHs were still highly proliferative as evidenced by a healthy nucleus with green fluorescence compared with red fluorescence under normal culture conditions. This result was confirmed by flow cytometry with Annexin V/PI staining. The number of apoptotic cells increased in the MSCs only group compared with the SMHs- MSCs group 12 hours after hypoxia (Fig. 2A,B).

The total Bcl-2 level in MSCs and SMHs-MSCs groups after hypoxia was significantly higher than that in control (MSCs hypoxia) group. The level of Bcl-2 in the hypoxic SMHs-MSCs group was higher than that in the hypoxic MSCs group (*P* < 0.05). There was an opposite trend with regards to the levels of Bax and caspase-3, such that protein expression decreased in the SMHs-MSCs group whereas their levels were increased in the MSCs group (*P* < 0.05). Therefore, SMHs can can induce changes in protein expression to minimize the effects of apoptosis (Fig. 2C).

Gene transcript expression levels of NKx2.5 and GATA-4 were significantly elevated in the SMHs-5-aza-MSCs group compared with the 5-aza-MSCs group after 2 weeks of incubation, as demonstrated by (Real-time Quantitative PCR Detecting System) qPCR (Fig. 2D). The expression of cardiac troponin T (cTnT) in the 5-aza + SMHs group increased significantly compared to the 5-aza only group. cTnT was not expressed in the PBS control group (without 5-aza). The MSCs induced by 5-aza in the SMHs formed clusters and generated cell junctions, which could be seen under phase contrast microscopy. cTnT and desmin were visible in the SMHs-5-aza-MSCs and 5-aza-MSCs groups, however, higher fluorescence intensity was observed in the SMHs-5-aza-MSCs group (Fig. 2D,E).

### Immunohistology studies *in vivo*

**Figure 3:**
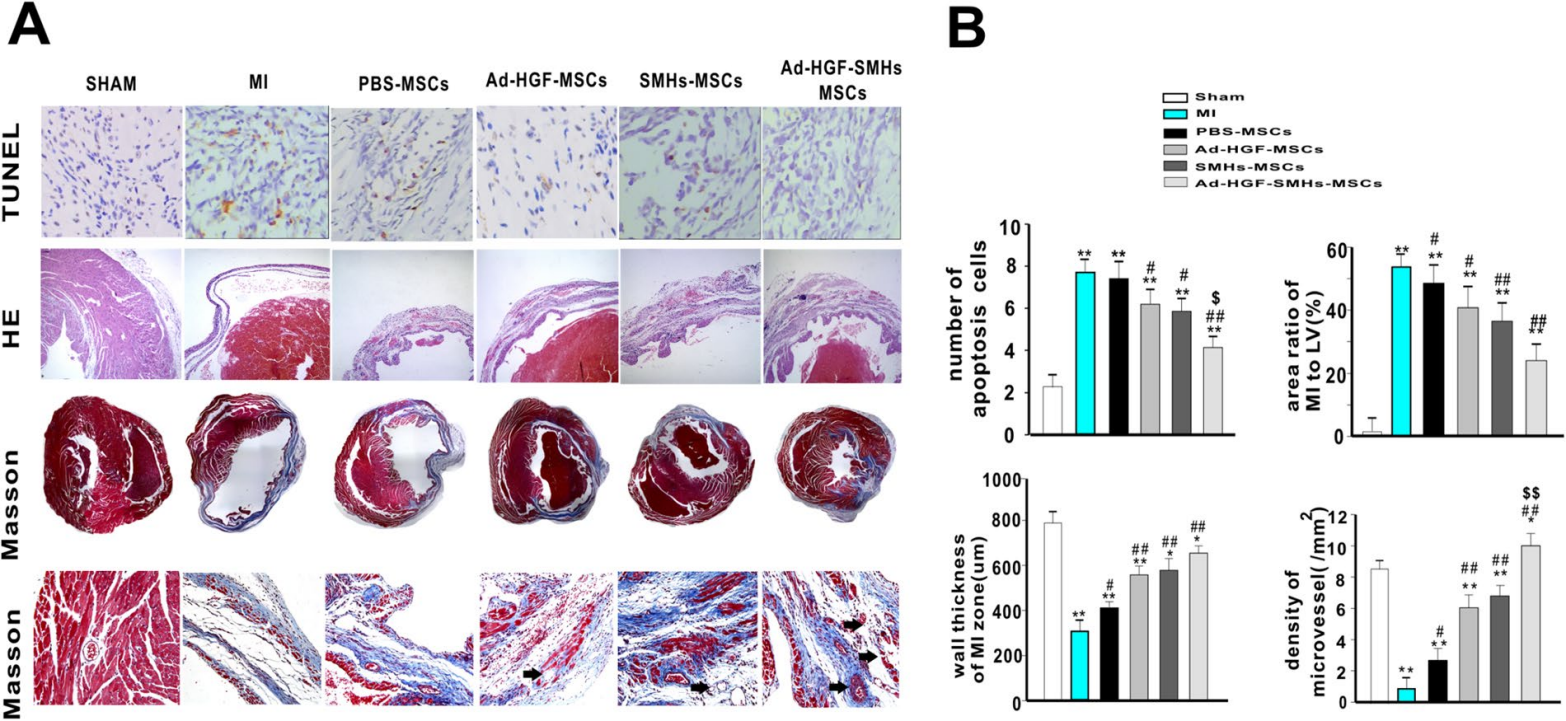
Immunohistochemistry staining *in vivo*. **(A)** In the peri-infarcted area apoptotic cells were detected and calculated by TUNEL assay. Photomicrographs of the LV tissue sections stained by H&E and Masson staining exhibited increased micro-vessel density, ratio of the MI area to LV%, and wall thickness in the MI zone. Magnification: 400x. (**B**) The graph shows the statistics of apoptotic cells, density of micro-vessel and ratio of the MI area to LV% as well as wall thickness in the MI zone. Data are mean ± SEM. *or **P < 0.05 or 0.01 *vs*. Sham; ^*#*^ *or* ^*##*^ *P* < 0.05 or 0.01 *vs*. group MI. ^$^or ^$$^ *P* < 0.05 or < 0.01 *vs*. SMH-MSCs and Ad-HGF-MSCs, n = 9~10.

TUNEL results indicated that the number of apoptotic cells around the area of MI was significantly reduced in the Ad-HGF-SMHs-MSCs groups compared with the MI group (*P* < 0.01) and the Ad-HGF-MSCs group (*P* < 0.05). Meanwhile, there was no statistical difference among the PBS-MSCs and MI groups. Additionally, the number of apoptotic cells declined significantly in the Ad-HGF-SMHs-MSCs group compared with the SMHs-MSCs and Ad-HGF-MSCs groups (*P* < 0.05) (Fig. 3A).

H&E-stained cross-sections of the myocardium indicated that the injection of SMHs with Ad-HGF-MSCs (23.9 ± 5.25%) significantly improved the the MI area-to-LV ratio when compared across other control treatment groups: MI (52.5 ± 4.13%), SMHs-MSCs (36.4 ± 5.84%), Ad-HGF-MSCs (40.7 ± 6.74%) and PBS-MSCs (48.5 ± 5.79%) (*P* < 0.05). Our study also evaluated vascular density using H&E staining of the ventricular wall thickness at the infarction zone. Ad-HGF-SMHs-MSCs (682 ± 45 μm) increased wall thickness greater than the other groups (MI: 313 ± 59 μm; PBS-MSCs: 417 ± 49 μm; Ad-HGF-MSCs: 557 ± 54 μm; and SHMs-MSCs: 576 ± 58 μm),consistent with the findings on echocardiography. Masson staining reflected the vascular density in the infarction zone. The rats injected with Ad-HGF-SMHs-MSCs had a remarkably higher vascular density (9.8 ± 1.32/mm^2^) compared to the Ad-HGF-MSCs (5.9 ± 1.02/mm^2^), SMHs-MSCs (6.8 ± 1.37/mm^2^), and PBS-MSCs (2.7 ± 0.53/mm^2^) groups (n = 9 ~ 10) (Fig. 3A).

**Figure 4:**
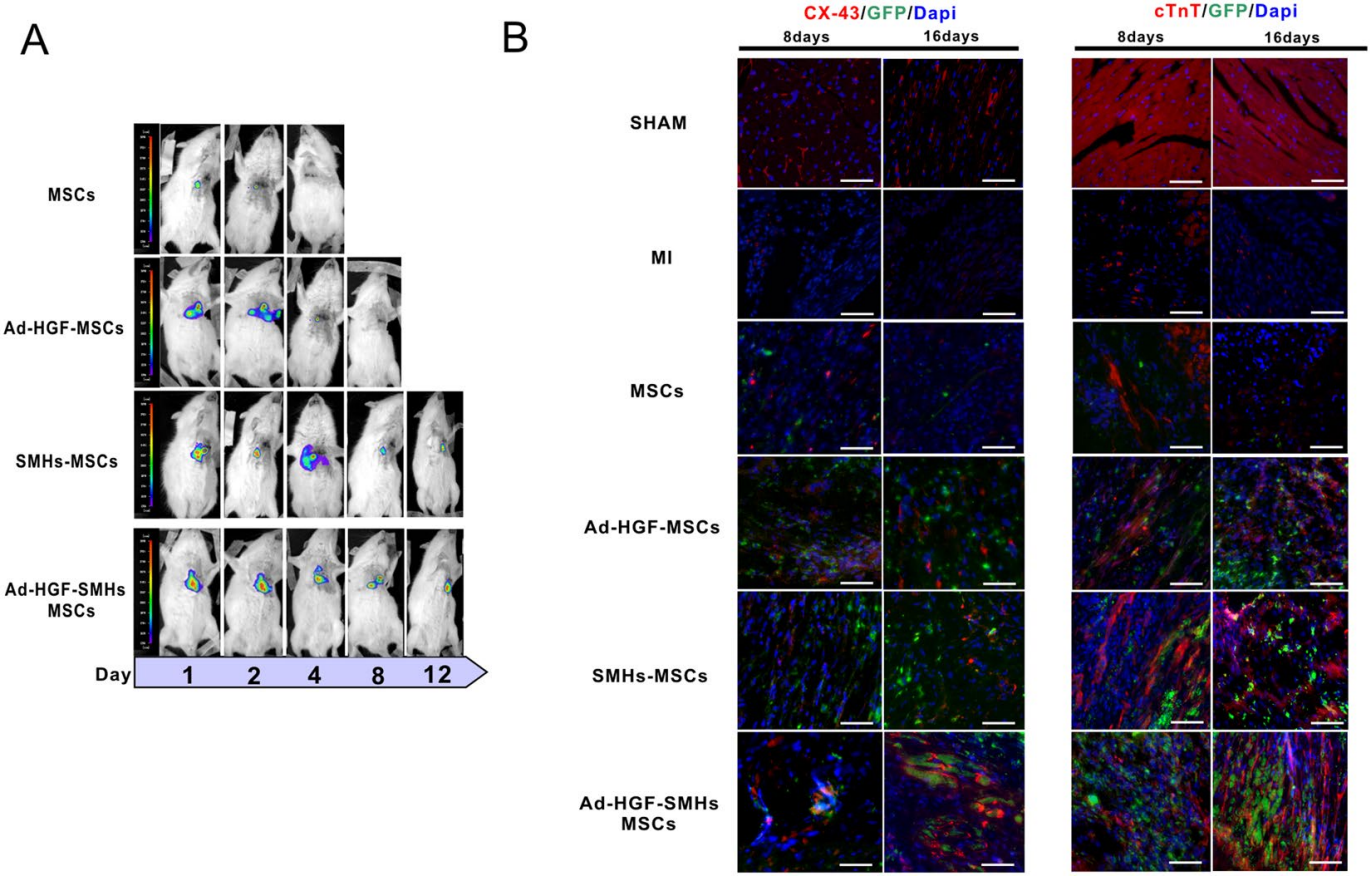
Immunofluorescence and bioluminescent imaging *in vivo*. (**A**) Retention and survival signals in transplanted cells were monitored by non-invasive bioluminescent imaging technology. (**B**) MSC retention and survival induced by 5-aza in each treatment group was measured by green fluorescence; The expression of cTnT and Cx43 in the 8days and 16days section were displayed as red fluorescence, Blue fluorescence: DAPI-stained nuclei n = 6. Magnification: 400x.

Image Pro Plus (IPP) 6.0 software analysis showed that higher blue (DAPI) and green fluorescence were found in the Ad-HGF-SMHs-MSCs and SMHs-MSCs groups compared to the Ad-HGF-MSC and other groups (*P* < 0.01, data not shown). SMHs significantly improved the retention and survival of Ad-HGF/MSCs after 16 days. The infarcted and surrounding area injected with Ad-HGF-SMHs-MSCs exhibited higher density of cardiac biomarkers including cTnT and Cx43 at 8 and 16 days (Fig. 4A). Some MSCs formed connections to the myocardium.

Non-invasive bioluminescent imaging was used to monitor the retention and survival of transplanted cells. Results indicated that the bioluminescent intensity of the Ad-HGF-SMHs-MSCs group was significantly increased compared with the Ad-HGF-MSCs group at day 2 (3.25 ± 0.26 × 10^6^ *vs*. 0.84 ± 0.13 × 10^6^ p/sec/cm^2^/sr; *P* < 0.05, n = 6) and at day 4 (2.72 ± 0.15 × 10^6^ *vs*. 0.34 ± 0.08 × 10^6^ p/sec/cm^2^/sr; *P* < 0.01, n = 6). Bioluminescent signal was also detected in the Ad-HGF-SMHs-MSCs and SMHs-MSCs groups from day 8 to day 12. In contrast, there was no signal observed in the Ad-HGF-MSCs group (Fig. 4B). Therefore, SMHs can promote stem cell retention and survival. MSCs-luc2+ bioluminescent *in vitro* imaging, correlation analysis, and quantitative data of photon emission for each animal and time-point are shown in Supplementary Material (Figure S8).

### Cardiac function

**Figure 5:**
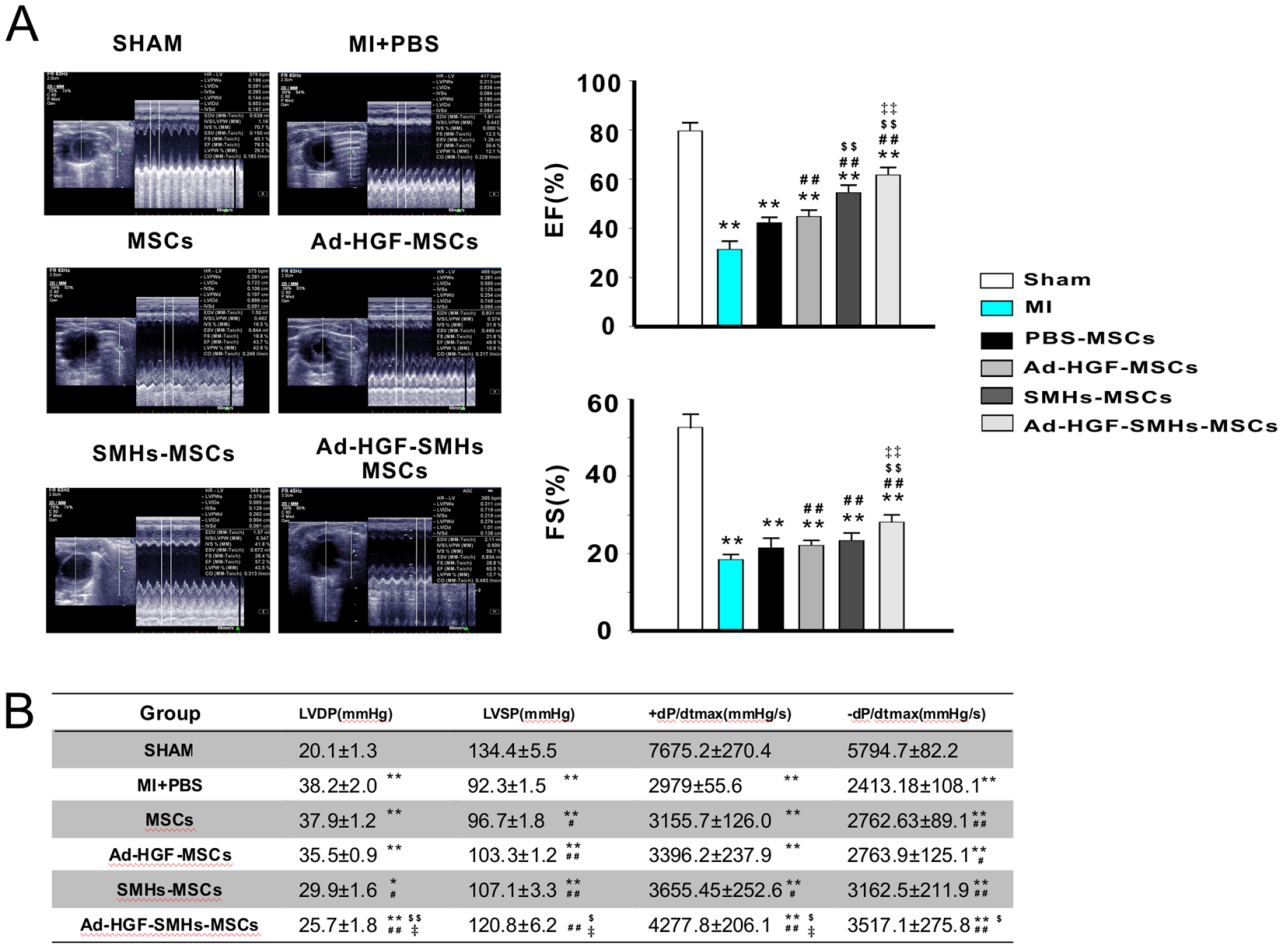
Improvement of cardiac function. (**A**) Representative pictures of echocardiography in each group and EF(%) and FS(%) statistics from bar graphs. (**B**) Table for cardiac hemodynamic monitoring. Data are mean ± SEM. *P < 0.05, **P < 0.01 *vs*. Sham; ^#^ *P* < 0.05, ^##^ *P* < 0.01 *vs*. MI + PBS; ^$^ *P* < 0.05, ^$$^ *P* < 0.01 *vs*. Ad-HGF-MSCs; ^‡^ *P* < 0.05 *vs*. SMHs-MSCs; n = 9~10. One-way ANOVA, then followed by post-hoc Tukey’s test for multiple comparisons.

Echocardiographic analysis of cardiac function was carried out 4 weeks post-surgery. Left ventricular ejection fraction (EF%) in MI (33.5 ± 4.9), MSCs (42.4 ± 2.0), Ad-HGF-MSCs (44.2 ± 2.6), SMHs-MSCs (52.3 ± 2.9), and Ad-HGF-SMHs-MSCs (62.1 ± 3.0) groups was significantly decreased (*P* < 0.01) compared to the sham group (79.7 ± 3.4). Additionally, we observed EF% improvement in the Ad-HGF-MSCs, SMHs-MSCs, and Ad-HGF-SMHs-MSCs groups compared to the MI group. Surprisingly, the Ad-HGF-SMHs-MSCs group showed the most effect among the treatment groups. Fractional shortening (FS%) was markedly elevated in the Ad-HGF-SMHs-MSCs group (28.2 ± 1.9) compared with the MSCs (21.5 ± 2.5), SMHs-MSCs (23.4 ± 1.9), and Ad-HGF-MSCs (22.2 ± 1.3) groups (*P* < 0.01) (Fig. 5A). Data from hemodynamic monitoring revealed that left ventricular diastolic pressure (LVDP) was significantly lower in the Ad-HGF-SMHs-MSCs group compared with the MI and other treatment groups. Meanwhile, LVSP was higher compared with the other groups (SHAM, PBS-MI, MSCs, SMHs-MSCs, and Ad-HGF-MSCs) (*P* < 0.01). Measurement of +dP/dtmax, an indicator of myocardial performance showed the same trend as left ventricular systolic pressure (LVSP), suggesting better myocardial contractility or overall function in the Ad-HGF-SMHs-MSCs group than the other groups (*P* < 0.01) (Fig. 5B).

## Discussion

The novelty of this study was the use of SMHs in stem cell transplantation for MI treatment. Specifically, we demonstrated that SMHs provided scaffold materials for cardiac tissue engineering. We also showed that Ad-HGF transfected MSCs reduce apoptosis and improve resistance to oxidative stress *in vivo*. We further demonstrated that SMHs carrying gene-modified MSCs change the microenvironment following MI, by enhancing cell survival and differentiation, reducing scar formation, and accelerating angiogenesis.

### SMHs: Cardiac tissue engineering and scaffold materials

Studies have shown that hydrogels with larger nano- or micro-structures are mechanically stroner and less flexible compared with SMHs. Conventional hydrogels may be less accommodating for cells embedded in these hydrogels to stretch compared with SMHs, and these cells have relatively lower viability and proliferative capacity^30,31^. Thus, challenges and limitations persist in the application of biomaterials.

Extracellular matrix (ECM) hydrogels are not eligible for tissue engineering as the result of low range stiffness relative to extremely high stiffness of myocardium. ECM hydrogels also have cytotoxic effects caused by glutaraldehyde. Therefore, ECM hydrogels may not be the best choice for cell-based application^32,33^. We designed SMHs and modified the properties, including elasticity, crystallinity, cross-linking density, and porosity of short peptide SMHs. This resulted in impactful results on MSC differentiation and engraftment capacity. Song *et al*. indicated that enhancement of cell therapy was attributed to mechanical stimulation that activated cell-surface receptors and subsequent signaling pathways^34^.

The SMHs used in this study was formed by the peptide ^D^FEFK^D^FEFKYRGD. As our previous reported^35^, the peptide ^D^FEFK^D^FEFKYRGD can form hydrogels which possess excellent histocompatibility and elasticity could be applied for the production of cell colony of *HeLa* cells. However, we found that the peptide ^D^FEFK^D^FEFK (without RGD) could not form a clear solution in pure water at pH of 7.4. Consequently, it can not form hydrogels. Thus, we were unable to encapsulate MSCs. The peptide ^D^FEFK^D^FEFKYRGD in the present study possesses an Arg-Gly-Asp (RGD) sequence. The RGD sequence is a motif in cell-surface integrins that facilitates ligand binding and can be used to modify the behavior of scaffold to promote cell adhesion and differentiation. Currently, an RGD-modified sequence is being used to treat inflammation, cardiovascular disease and metastatic tumors^36,37^. Previous studies have demonstrated that the RGD structural domain regulates integrin αvβ3 expression in the early stage of heart development, and promotes the differentiation of MSCs into cardiac muscle cells^38^. On the other hand, SMHs provide the chemical microenvironment to reduce toxicity of 5-aza. Our hydrogel SMHs was prepared without any chemical triggers and could be gelled at physiological conditions with thixotropic (time-dependent shear thinning effect) properties, allowing it to be considered an injectable. Histocompatibility and homeostasis tests showed that the injection of 1% SMHs did not lead to a severe inflammatory response. The *in vivo* biocompatibility and biodegradation tests of the hydrogel indicated that our hydrogel was suitable as a scaffold material for cardiac tissue engineering and regenerative medicine.

### Ad-HGF transfected MSCs reduce apoptosis and improve oxygen resistance ability in SMHs

HGF stimulates angiogenesis, limits scar formation, and reduces fibrosis^39–41^. HGF can reduce apoptosis of myocardial cells *in vivo* and *in vitro* ^42^. In this study, adenovirus was chosen as the vehicle to to increase forced expression of HGF in MSCs. Consistent with previous studies^43^, we found that after 48 hours of Ad-HGF infection, HGF expression was increased at both mRNA and protein levels in MSCs, and reached a maximum value of 121.4 ng/mL at 3 weeks. Madonna *et al*. reported that HGF could protect against cell apoptosis caused by many stimuli^44^. It is also well known that Bcl-2 and Bcl-xL play essential roles in anti-apoptosis. Bax apoptotic sub-family (Bax and Bak) contribute to their oligomer conformation, which responsible for permeabilizing membrane of the mitochondrion and resulting in apoptosis by activating executioner caspases. Thus, Bcl-2 and Bax are crucial in modulating apoptosis through upstream regulation of caspase-3 activation. Hypoxic-ischemic-induced apoptosis contributed to decreased expression of Bcl-2 and increased expression of Bax^45^. In our experiment, MSCs transfected with Ad-HGF after 12 hours of hypoxia showed overexpression of Bcl-2 and decreased expression of Bax and caspase-3. This result is consistent with previous reports^46^. In contrast, HGF-mediated c-MET activation increased angiogenesis and reduced fibrosis. The colon cancer 1 (MACC1) gene is an essential pro-metastatic factor in human colon cancer. MACC1 has been demonstrated to activate the HGF/c-MET pathway, which results in multiple cellular responses regulating proliferation, cell migration and ECM breakdown^47^. Notwithstanding, the mechanism by which HGF in MSCs ameliorates apoptosis and fibrosis, as well as increase angiogenesis, is still unclear and needs to be studied in the future.

Previous research has showed that endogenous HGF is blocked in occlusion-reperfusion studies, and, consequently, the infarct size and mortality rate are increased significantly. As a pharmacological delivery system, hydrogel or gelatin can improve the effect of the HGF released by stem cells^28,29^. The results of our study also indicate that SMHs work with HGF to exert excellent therapeutic effects on systemic disease requiring the participation of stem cells. SMHs have great potential for tissue engineering^48^ and drug delivery^49^. Prior research reported that self-assembling peptide hydrogels encapsulated with curcumin could serve as injectable delivery vehicles^50^. To some extent, injectable SMHs provide sustained and controlled HGF release in the MI zone, as well as retention and efficacy, promoting reduced apoptosis and improved oxygen resistance ability.

### SMHs carrying gene-modified MSCs improve MI

We observed that SMHs mediated expression of apoptosis-related proteins. The MSC group showed an overexpression of Bcl-2, Bax and caspases-3 following exposure to hypoxia for 12 hours,indicating the presence of cell apoptosis^51–53^. In the SMHs-MSCs group, Bax and caspases-3 protein levels were downregulated, accompanied by an overexpression of Bcl-2. Thus, SMHs can improve cell survival and resistance to a hypoxic microenvironment. Notably, SMHs served as a 3D platform for cell culture, which regulated apoptosis as evidenced by Western blot, CCK-8 and MTT via following mechanisms: (1) Mechanical stimulation of 3D microenvironment activated intracellular signaling pathways to directly improve MSC survival; (2) binding of specific ligands in SMHs with surface receptors on MSCs to regulate downstream signaling pathways, and (3) exertion of paracrine effects on cell survival mechanisms under anoxic conditions.

NKx2.5, GATA-4 and desmin are the early differentiation markers in cardiac progenitor cells^54,55^. Cx43, a gap junction protein, ensures electrical and metabolic coupling between cardiomyocytes and coordinates their contractility^56^. Yang *et al*. found that genes indicative of MSC differentiation into cardiomyocytes induced by 5-aza was verified by qPCR, Western blot and immunohistology^57,58^. In our study, the expression of NKx2.5 and GATA-4 was up-regulated in SHMs-5-aza-MSCs compared to the 5-aza MSCs control. Moreover, specific myocardial proteins, such as cTnT and Cx43, were expressed at higher levels in SHMs-5-aza-MSCs compared with the 5-aza MSCs control *in vitro*, and in Ad-HGF-SMHs-MSCs compared with others *in vivo*. The TUNEL results demonstrated that SMHs protected against cell apoptosis induced by MI. As evidenced by immunostaining, we found that SMHs improved cell retention and survival. DAPI and GFP fluorescence were stronger in Ad-HGF-SMHs- MSCs compared to PBS-Ad-HGF-MSCs. Additionally, we hypothesized that MSCs in PBS were vulnerable in the heart but SMHs provided a support to protect the cells.

Echocardiographic analysis showed that the Ad-HGF-SMHs-MSCs group had increased LV wall thickness, systolic function, and LV chamber dimension compared with the PBS and Ad-HGF-MSCs control groups. Most of the angiogenesis and microvessels were found in the peri-infarcted and adjacent areas in the Ad-HGF-SMHs-MSCs group 4 weeks after transplantation. We propose that this result is due to the following mechanisms: (1) SMHs serving as cell scaffold to provide the microenvironment that is similar to *in vivo* conditions; (2) enhanced tenacity of SMHs affecting the elasticity of scar tissue by increasing the thickness of infarcted tissue to improve tissue compliance and heart function^59^; (3) SMHs ameliorating angiogenesis to increase cell survival; (4) RGD in SMHs interacting with MSCs to protect the cell from anoikis^60^; (5) SMHs stimulating heart development-related genes in the early phase mediated by RGD; and (6) SMHs controlling Ad-HGF release, therefore prolonging the effect of HGF to improve MSCs survival, differentiation and angiogenesis.

### Limitations and future research

Although our study confirmed the apparent value of SMH therapy, This study has a number of limitations with Ad-HGF-MSCs in experimental MI, the pathways by which SMHs affect MSCs proliferation, survival, and differentiation are still unknown. In the current literature, most studies have focused on studying the properties of biomaterial and its apparent effects, but there is little focus on the underlying mechanisms. In order to determine the mechanisms of SMHs’ systematic effect on different cells, we will devote efforts to study the relative effect of peptide reactive group biomaterial on cell surface protein kinases or antigenic determinants in our future research.

## Summary

HGF can effectively reduce cell apoptosis and promote growth and proliferation of MSCs. Chemical and physical factors provided by the SMHs promote cell growth, proliferation and differentiation. Delivery of MSCs by SMHs improved the microenvironment of transplanted stem cells. A multitude of mechanisms associated with SMHs favors stem cell adhersion, survival and direction differentiation of myocardial cells, leading to reduced scar area, enhanced angiogenesis and an improvement in myocardial function.

## Materials and Methods

### Preparation of SMHs

Hydrogels were produced through non-covalent interactions (π - π, hydrogen bond, ionic bond, *etc*.). At Nankai University, we previously designed a novel small molecule, ^D^FEFK^D^FEFKYRGD, which can form hydrogels in physiological environments^35^. The hydrogel characterization is shown in the Supplement (Figs S2–4). The peptide was prepared by solid phase peptide synthesis (SPPS). 10 mg SPPS was dissolved in 0.4 mL H_2_O_2_, then the pH was adjusted to 7.4 by sodium carbonate, and mixed with 0.5 mL phosphate buffered saline (PBS). The vessel was placed upside down to promote formation of a jellylike colloid. The examination of hydrogel biocompatibility were conducted in previous work^31^.

### MSC origin and maintenance

MSCs were obtained from Cyagen Biosciences Inc, (USA) and cultured in DMEM supplemented with 10% FBS, streptomycin (100 U/mL), and penicillin (100 U/mL). Detection of markers CD34, CD45, CD11b/c was done by flow cytometry analysis and multi-potential differentiation. All MSCs used in this study were used by passage 5 (P5). MSC culture and passage were performed according to instructions from Cyagen Biosciences Inc. MSCs in P5 were cultured for 7 days before injection. 5-aza (4 μmol/L) was added to DMEM on day 4 and incubated with MSCs for 24 hours. All MSCs were then cultured in serum-free DMEM. The respective groups were cultured in anoxic conditions for 12 hours: 94% N_2_, 1% O_2_ and 5% CO_2_.

### Transfection of MSCs and cell proliferation and viability analyses

Lentivirus infection and reporter gene pre-construction of lentiviral vector expressing firefly luciferase2 (Luc2) were prepared by AdmaxTM Kit D (Microbix, Canada). Luciferase fragment was amplified by PCR. Each qPCR reaction system volume was 50 µl, including: 5 x Prime STAR Buffer (10 µl), 10 mmol/L dNTP (1 µl), 5 µmol/L Primer (4 µl), DNA template (3 µl), Prime STAR enzyme (0.5 µl), and 1% H_2_O_2_ (31.5 µl). The PCR parameters were: 98 °C for 30 sec, 98 °C for 10 sec, 62 °C for 5 sec, 72 °C for 80 sec, for 32 cycles, and 72 °C extension 5 min. Luc2-F: ttcgaaAGCCACCATGGAAGATGCCA, Luc2-R: gcggccgcTTACACGGCGATCTTGCCG. We used QIAquickTM Gel Extraction Kit, Tailing Kit, pMD18-T Kit and QIAGEN Kit (Qiagen, Germany) to establish the pLenti- luc2+, following transfection of HEK293 cells and collection and purification of lentivirus. GFP-Adenovirus was purchased from Zhengyang (Beijing). Plasmids pAd-HGF, pDC316, and pBHGloxΔE1, 3Cre were donated by Dr. Li Ma (Institute of Molecular Immunology, SMU, China). We set up a recombinant Ad-GFP-vector with HGF gene and transfected it into MSCs. The expression level of HGF mRNA in MSCs was checked by quantitative PCR. The expression of HGF protein was confirmed by enzyme-linked immunsorbent assay (ELISA) (Sigma) and the effect of cell supernatant on MSC growth was tested by MTT. The effect of hypoxia on HGF expression was measured by Annexin V/ propidium iodide (PI) flow cytomety and Western blot. To monitor tolerance to 12 hours hypoxia in MSCs that were either gene-modified or encapsulated in SHMs, MSCs were divided into subgroups: PBS-MSCs, Ad-GFP-MSCs, Ad-HGF-GFP-MSCs, and SMHs-MSCs in either normal culture or hypoxia.

### Animal model and injection of biomaterial with MSCs

The study was performed in accordance with the Guide for Animal Care and Use Committee of Southern Medical University, and National Institutes of Health Guidelines on the Use of Laboratory Animals (NIH publication NO. 85-23, revised 1996). The experimental protocols were also approved by Animal Care and Use Committee of Southern Medical University. Male Sprague-Dawley rats (9–10 weeks old) weighing 200 ± 20 g were divided into the following subgroups: sham, MI, and therapeutic groups (PBS-MSCs, Ad-HGF-MSCs and Ad-HGF-SMHs-MSCs) (n = 9 to 10 animals per group). All groups were fasted for 12 hours and given water before surgery. Rats were anesthetized using 10% chloral hydrate (0.3 mL/100 g) and prior to tracheal intubation. ‘After thoracotomy, muscle layers were separated, pericardial membrane was removed, and the left auricle was exposed. The left coronary artery, as tied off about 2 mm below the left auricle using a 6-0 silk suture at a depth of 0.5 mm to form the experimental MI. The same procedure was followed in sham group except the artery was not tied. ST elevation was detected by RM6240BD-ECG. Penicillin was administered for three days post-surgery. Seven days post-surgery, thoracotomy and tracheal intubation were carried out again. MSCs were incubated with DAPI for 15 min before injection. The MSCs (5 × 10^6^) were suspended in SMHs and injected around the area of MI with a 29-gauge needle.

### Bioluminescent imaging, histological and immunohistochemical staining

Non-invasive bioluminescent imaging was used to measure MSC retention. The rats in the MSC group and therapy groups were anaesthetized by 10% chloral hydrate, underwent coronary artery ligation, and were injected with MSCs or Ad-HGF-MSCs infected with lentiviral-luciferase2 (luc2) with or without SMHs near the MI zone (5 × 10^6^, 150 µl, n = 8). The rats were administrated 150 µl D-luciferin (15 mg/mL) by intraperitoneal injection. The signal was captured by imaging technology (Night OWL II LB983 NC100, Berthold) and analyzed after 1 to 14 days.

To assess the degree of apoptosis in the peri-infarct areas, the left ventricle (LV) tissue was sampled 2 weeks post-surgery. LV samples were fixed in 4% paraformaldehyde, embedded with liquid paraffin, and cut into 5 µm thick sections. The sections were treated according to instructions in the One Step TUNEL Apoptosis Assay Kit (Takara). Samples were imaged using a fluorescence microscope. Enumeration was performed counting the number of positive cells in five views per sample and five slides per group.

LV samples were fixed in 10% neutral buffered formalin, embedded with paraffin, and sectioned. Myocyte cross-sectional area was used to evaluate the proportion of MI in the LV. Briefly, sections were deparaffinized with xylene and stained with H&E. Sections were stained with a Masson trichrome kit (Baso, BA4079) according to the manufacturer’s instructions. The stained sections were examined under a light microscope (Nikon ECLIPSE Ti-U). The area of the MI, cross sectional area of the LV chamber, and the thickness of the wall were measured by Image Pro Plus 6.0 (IPP, Media Cybernetics, Carlsbad, CA).

Frozen sections of rat heart (n = 6) were fixed in acetone and perfused with PBS twice, followed by permeabilization with 0.1% Triton X-100 and blocked with normal goats serum for 20 min. Cardiac troponin (cTnT) and connexin 43 (Cx43) antibodies (SantaCruz, USA) were diluted in blocking solution (l:500). Cy5-labeled goat anti-mouse IgG (l: 500, Chemicon, USA) was used for incubation in the dark. We observed positive cells by determining the green and red fluorescence using fluorescence microscopy.

### Evaluation of cardiac function

At 4 weeks post-surgery, rats were weighed and anesthetized with chloral hydrate before examination. Transthoracic two-dimensional guided M-mode echocardiography was conducted using an IE33 echocardiographic system (Philips Medical Systems) outfitted with the 15-MHz (s12) transducer, as we previously described^61^. The parameters of the systolic function fractional shortening (FS) and LV ejection fraction (EF) were obtained by the improved Simpson method. Hemodynamic parameters were measured according to the following protocol. The animals were anesthetized with 10% chloral hydrate solution on the same day that the cardiac ultrasonography examination was performed. The right common carotid was separated and the distal end was ligated with thread while the proximal end was occluded. The multichannel animal physiological signal acquisition processing system was applied to record the LVP curve LVEDP, and +dp/dtmax was also recorded by the PowerLab system.

### Quantitative real-time qPCR and Western blotting

Cell RNA was extracted by Trizol. The relative expression level of NKx2.5 and GATA-4 was analyzed by real-time PCR. We used SYBR Green combined with DNA emitted fluoresce. Data were analyzed using the 2^−ΔΔCT^ to calculate the CT value.

Nkx2.5-F: 5′-GAGCCTACGGTGACCCTGACCCAG-3′;

Nkx2.5-R: 5′-TGACCTGCGTGGACGTGAGCTTCA-3′;

GATA-4-F: 5′-CATGCTTGCAGTTGTGCTAG-3′;

GATA-4-R: 5′-ATTCTCTGCTACGGCCAGTA-3′;

GAPDH-F: 5′-CCCCCAATGTATCCGTTGTG-3′;

GAPDH-R: 5′-TAGCCCAGGATGCCCTTTAGT-3′;

Relative protein expression level of Bcl-2, Bax and caspase-3 was analyzed by Western blot. We chose the Bio-Rad Dc Protein Assay Reagent to determine protein concentration. Briefly, protein samples were separated by sodium dodecyl sulfate polyacrylamide gel electrophoresis (SDS-PAGE) and transferred to polyvinylidene difluoride (PVDF) membranes. PVDF membranes were probed with cTnT antibody (Abcam, 1:300), Bcl-2 antibody (Invitrogen, 1:500), caspase-3 (Invitrogen, 1:500), and Bax (Invitrogen, 1:500). The antibody-antigen complexes were detected using the ECL PLUS kit (Bio-Rad). The protein bands were analyzed with Image Pro Plus 6.0.

### Statistical analysis

Values are expressed as means ± standard error of the mean (SEM). One-way analysis of variance was used to analyze variations between the means of groups; SPSS 17.0 (SPSS Inc., Chicago, IL, USA) was utilized for analysis. Significant values are defined as P < 0.05.

A post hoc t-test (Newman–Keuls) was conducted in the method of S-N-K following the one-way ANOVA in SPSS.

## Electronic supplementary material

supplementary information
