# Supplementary material for: Treatment of Myocardial Infarction with Gene-modified Mesenchymal Stem Cells in a Small Molecular Hydrogel
Source: Sci Rep. 2017 Nov 20;7:15826. doi: 10.1038/s41598-017-15870-z (PMC5696474; doi:10.1038/s41598-017-15870-z)

**Treatment of Myocardial Infarction with Gene-modified Mesenchymal Stem Cells in a Small Molecular Hydrogel**

**Running title: Combined SMHs and Gene-modified MSCs in MI treatment**

Zhiye Wu^1,2#^, Guoqin Chen^3#^, Jianwu Zhang^4^, Yongquan Hua^1,2^, Jinliang Li^3^, Bei Liu^5^, Anqing Huang^1^, Hekai Li^1,2^, Minsheng Chen^1,2*^, Caiwen Ou^1,2*^

^1^ Department of Cardiology, Heart Center, Zhujiang Hospital, Southern Medical University. Guangzhou 510280, China

^2^ Guangdong Provincial Biomedical Engineering Technology Research Center for Cardiovascular Disease, Zhujiang Hospital, Southern Medical University. Guangzhou 510280, China

^3^ Cardiovascular Medicine Department of Central Hospital of Panyu District, Guangzhou 510280, China

^4^ Department of Cardiology, Nanfang Hospital, Southern Medical University, Guangzhou 510515, China.

^5^ Department of Cardiology, Shanghai general hospital. Shanghai 200000, China

^#^These authors contributed equally to this work and should be regarded as co-first authors.

***Corresponding author:**

Minsheng Chen

Zhujiang Hospital, Southern Medical University,

Guangdong Provincial Center of Biomedical Engineering for Cardiovascular Diseases, NO.1023,Shatai Nan Road, Guangzhou 510280, P. R. China.

Email: gzminsheng@vip.163.com;

Tel. /Fax: +8602061648001.

Caiwen Ou ,

Zhujiang Hospital, Southern Medical University,

Guangdong Provincial Center of Biomedical Engineering for Cardiovascular Diseases,

NO.1023,Shatai Nan Road,Guangzhou 510280, P. R. China.

Email: 1284572007@qq.com; Tel/Fax: +8602061648001.

**Supplementary Material**

Polymer hydrogels are utilized universally in all fields of engineering. We focused on the peptide-based hydrogel based, and in particular the small molecule hydrogel. In collaboration with Prof. Yang in Nan Kai University, we developed a series of supra hydrogel that have the following advantageous properties: a. harmless to humans with excellent bio-compatibility and degradability; b. mimic [extracellular](javascript:void(0);) [matrix](javascript:void(0);); c. adopted disulfide bond for reduction-induced hydrogel formation ; d. 3D culture drug delivery , hepatic regeneration and wound healing.

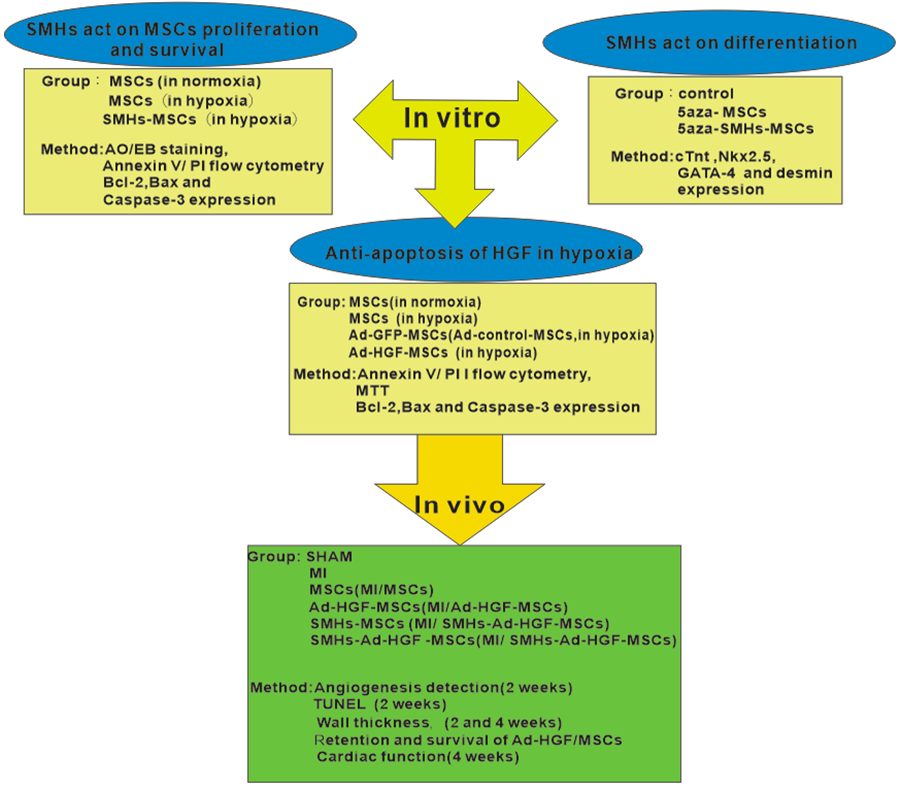


**Figure S1.** Model of experimental details (e.g. *in vivo* or *in vitro*, different groups, sample size, and study time points.)


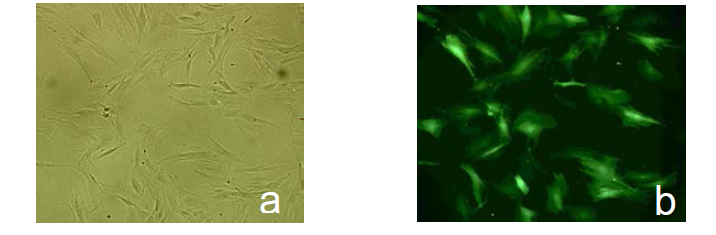


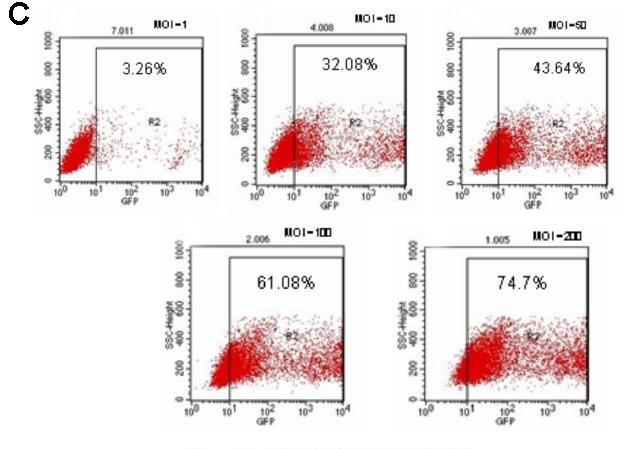


**Figure S2.** Detection of Ad-HGF transfection efficiency. Morphologic characteristics and fluorescent intensity was determined by fluorescence microscopy (a,b), which demonstrated that the expression of GFP increased with the increment of multiplicity of infection(MOI) and time, MOI increased under different gradients of 1,10,50,100 and 200. 48 hours was the strongest timepoint, after which the intensity remained invariant as time went on. c. Flow cytometry was used to measure the transfection efficiency of recombinant Ad- HGF. The value was between 3.26% and 74.7%; the optimum MOI was 200 and the highest transfection ratio was obtained 48 hours later.


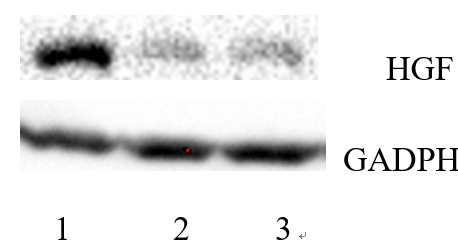


**Figure S3.** Western blot to measure HGF levels in MSCs 1: Ad- HGF; 2: Ad-GFP; 3. Control.


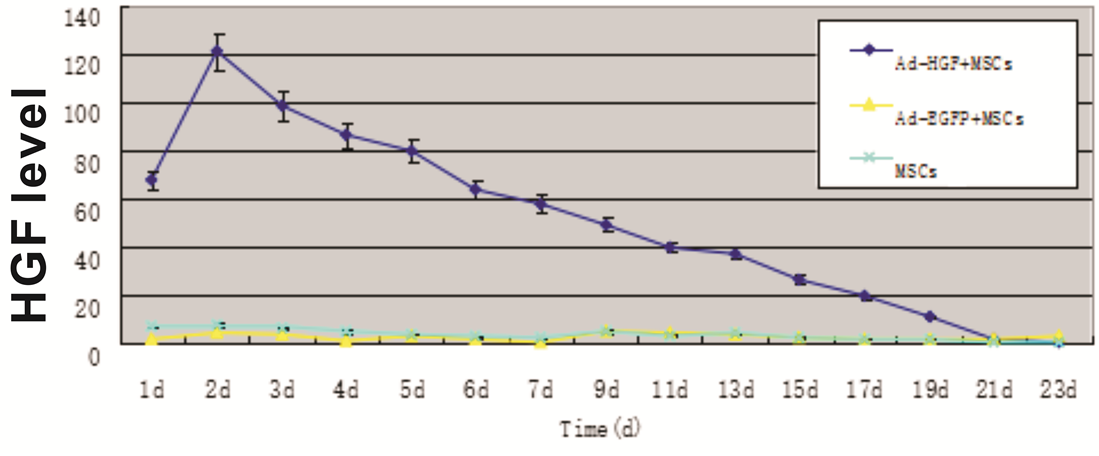


**Figure S4.** ELISA analysis. The level of HGF in cell supernatant peaked (121.4ng/ml) after 48 hours transfection and was continuously released for 23 days.


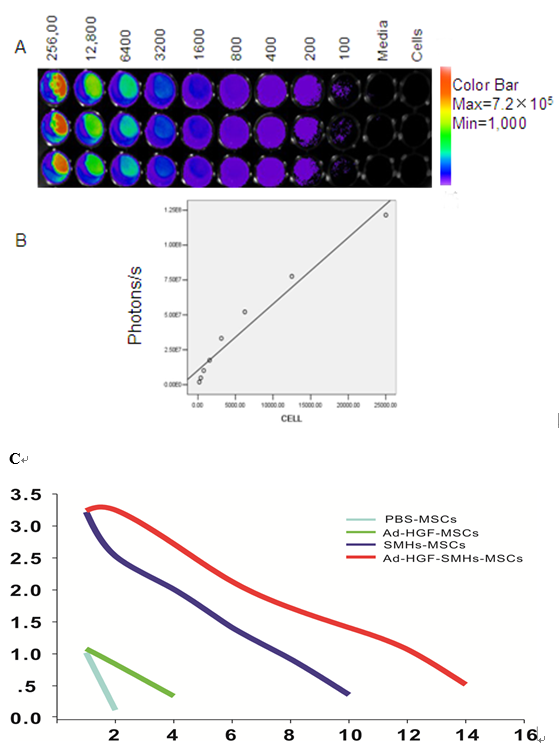


**Figure S5.** A, MSCs-luc2+ bioluminescent imaging *in vitro* and correlation analysis. Fluorescence intensity positive correlated with cell population, R^2^ = 0.96. B, minimum cell number for detection was 100. C, Quantitative data of photon emission for each animal and time-point.


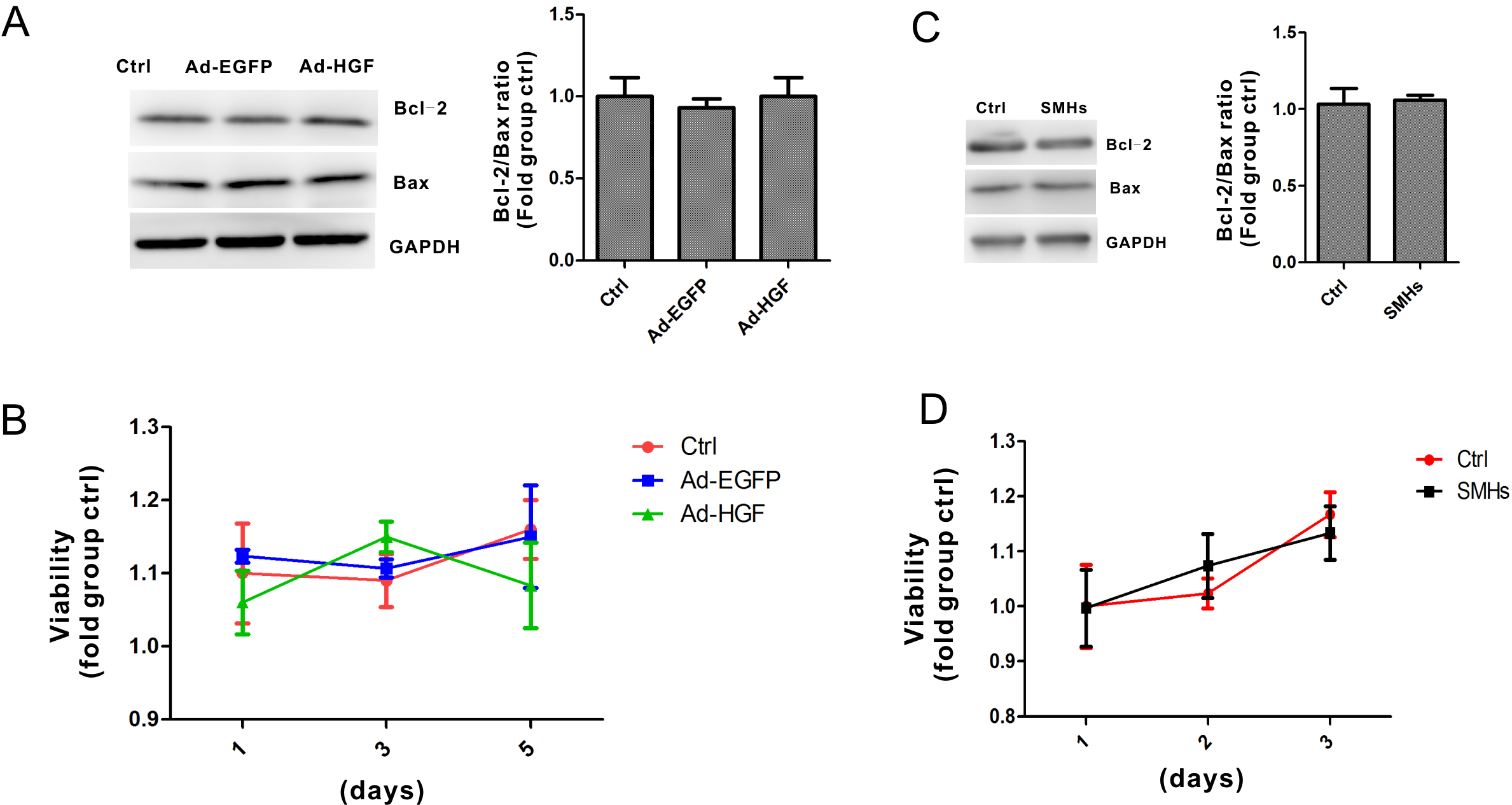


**Figure S6.** Fig.S6. Effect of Ad-EGFP and SMHs on apoptosis and cell viability in normoxia. A and C, Western blot analysis and quantification of Bcl-2 and Bax expression in MSCs. B and D. MTT curve for analyzing the viability of MSCs.


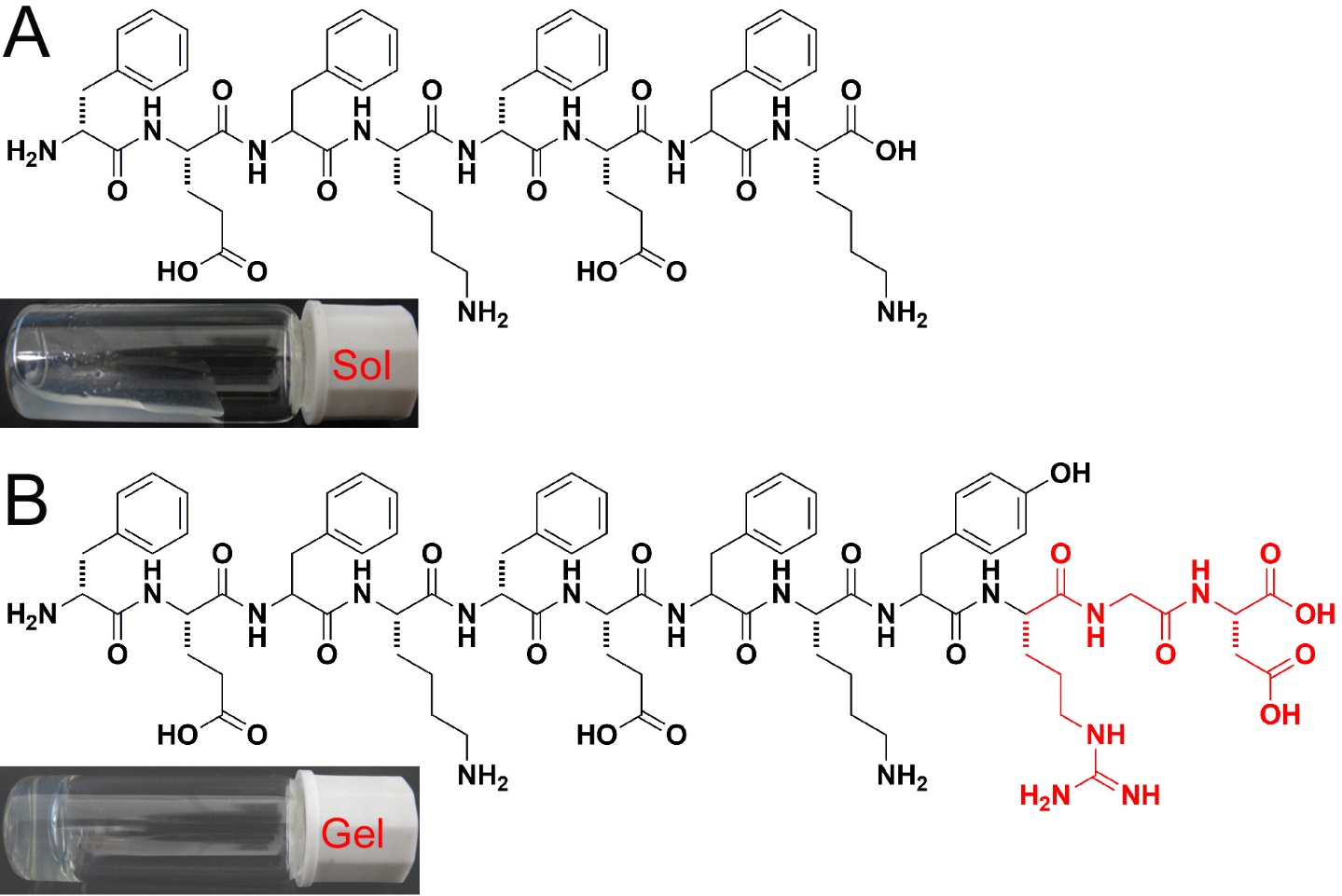


Figure S7. The chemical structure of ^D^FEFK^D^FEFK. Insert：the optical image of a PBS solution of ^D^FEFK^D^FEFK (1 wt%, pH = 7.4). B. The chemical structure of ^D^FEFK^D^FEFKYRGD. Insert：the optical image of a PBS solution of ^D^FEFK^D^FEFKYRGD (1 wt%, pH = 7.4).

**Western Blot gel exposure of figure1,2：**


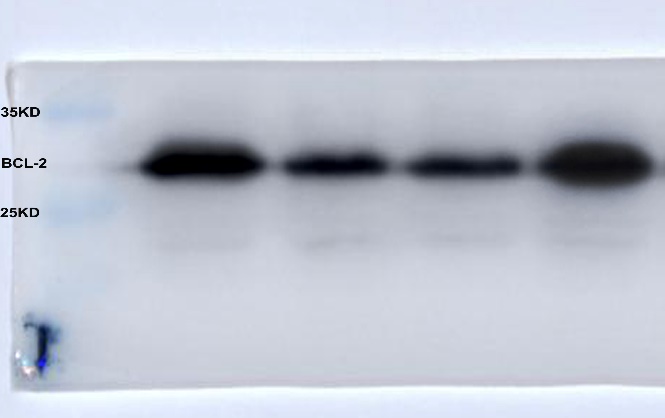

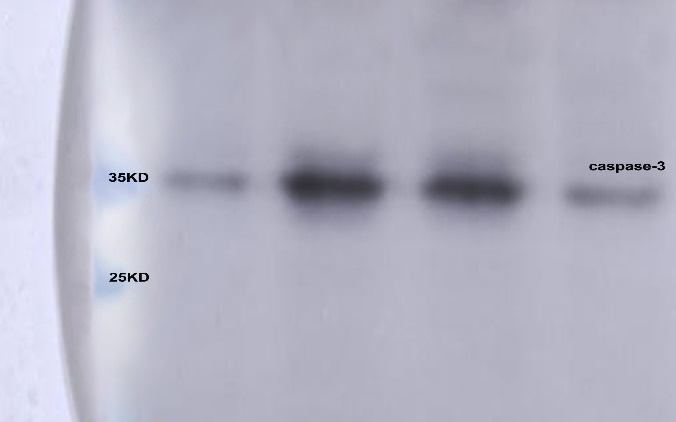


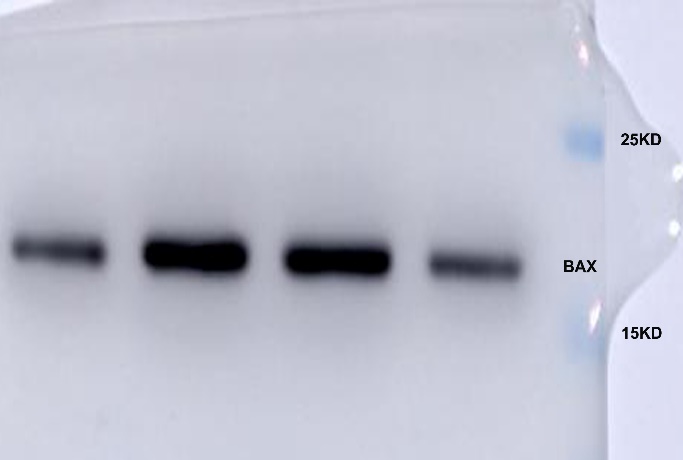

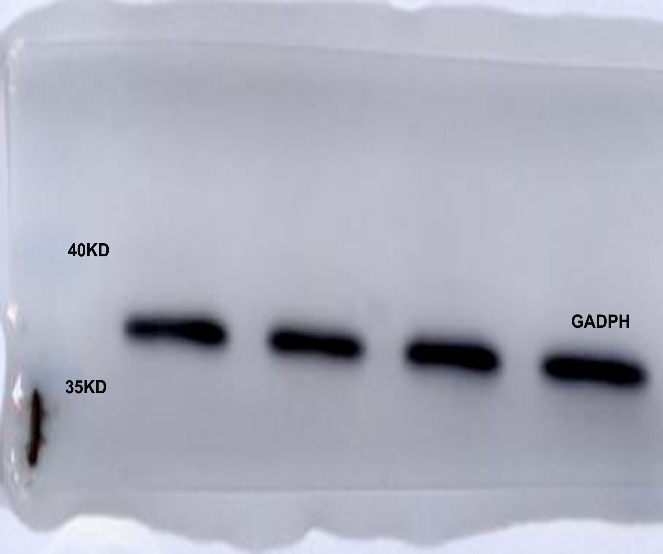


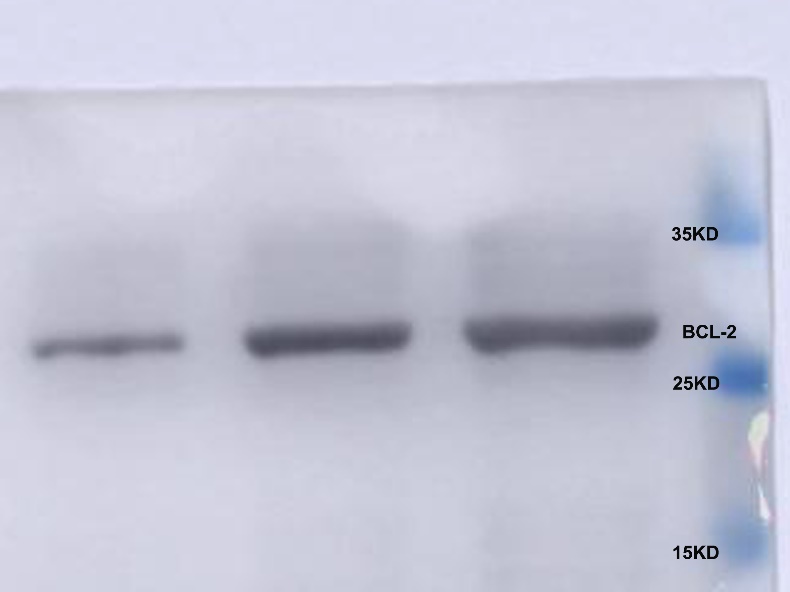

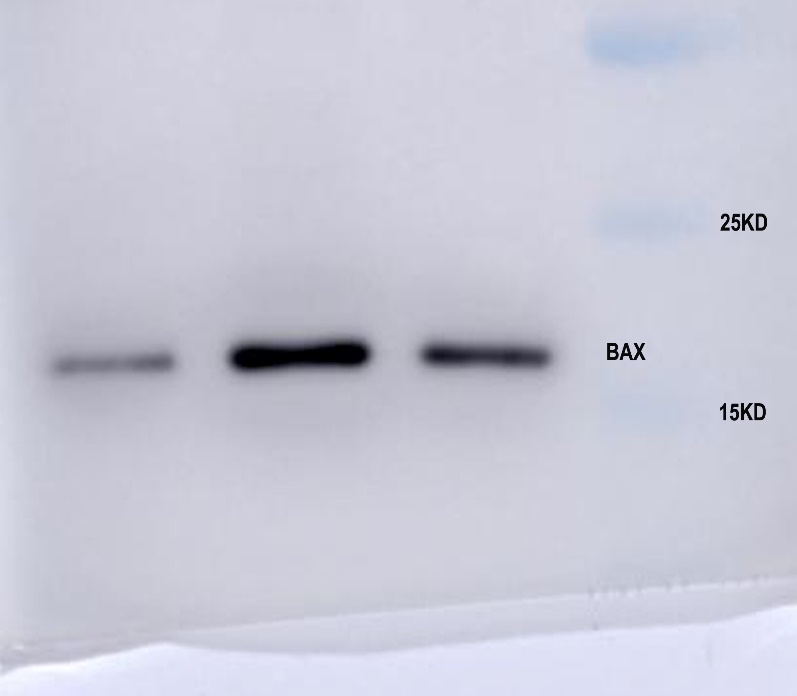


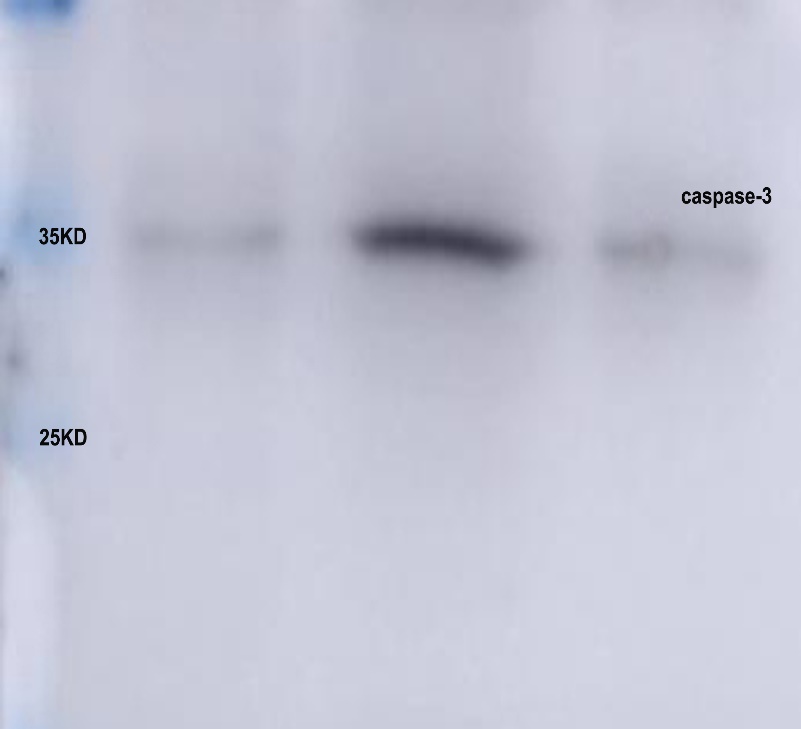

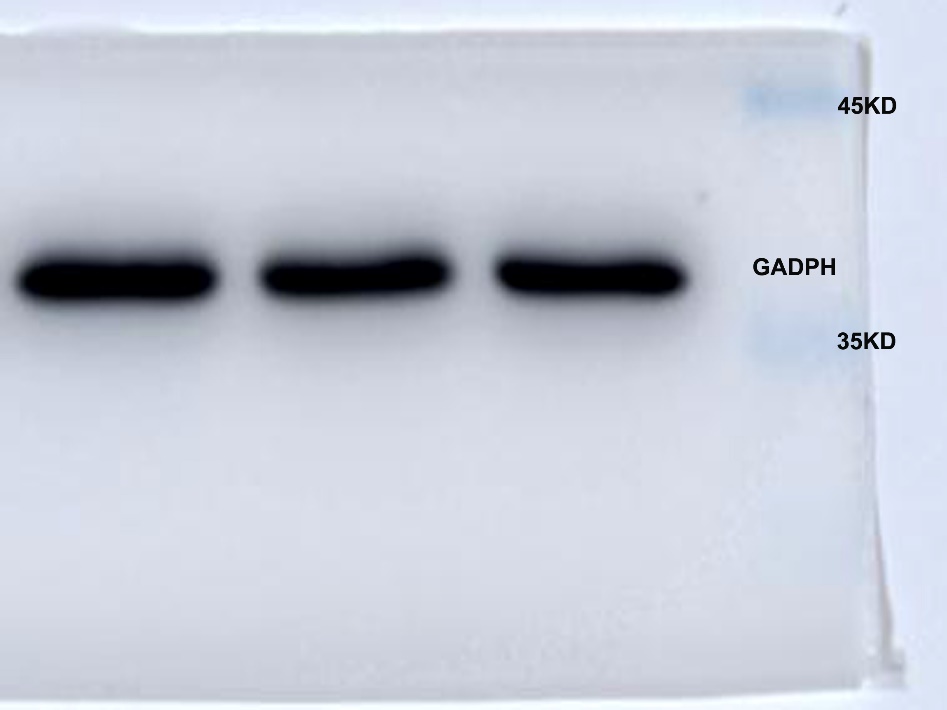


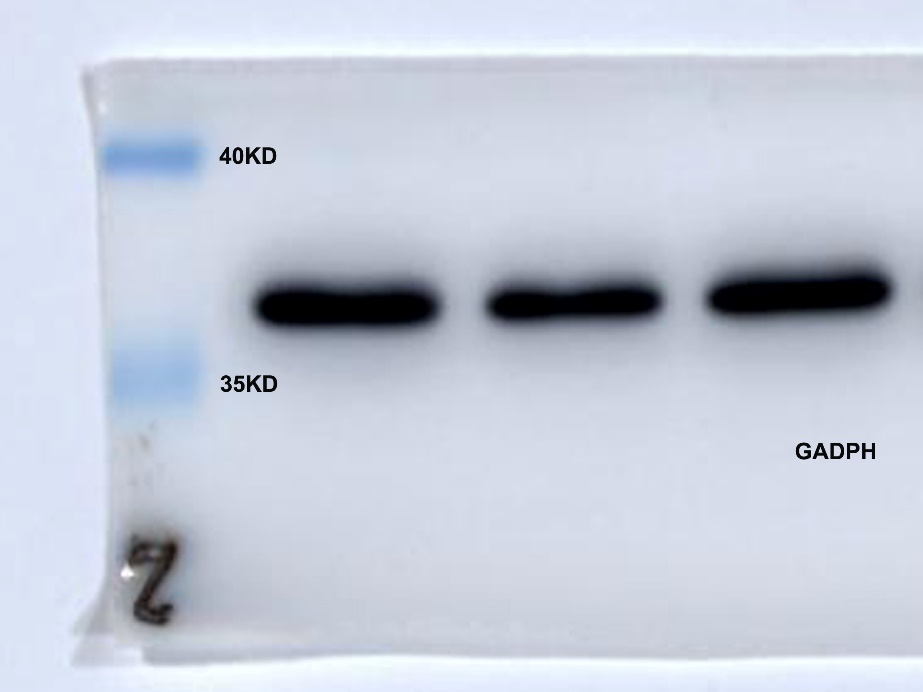

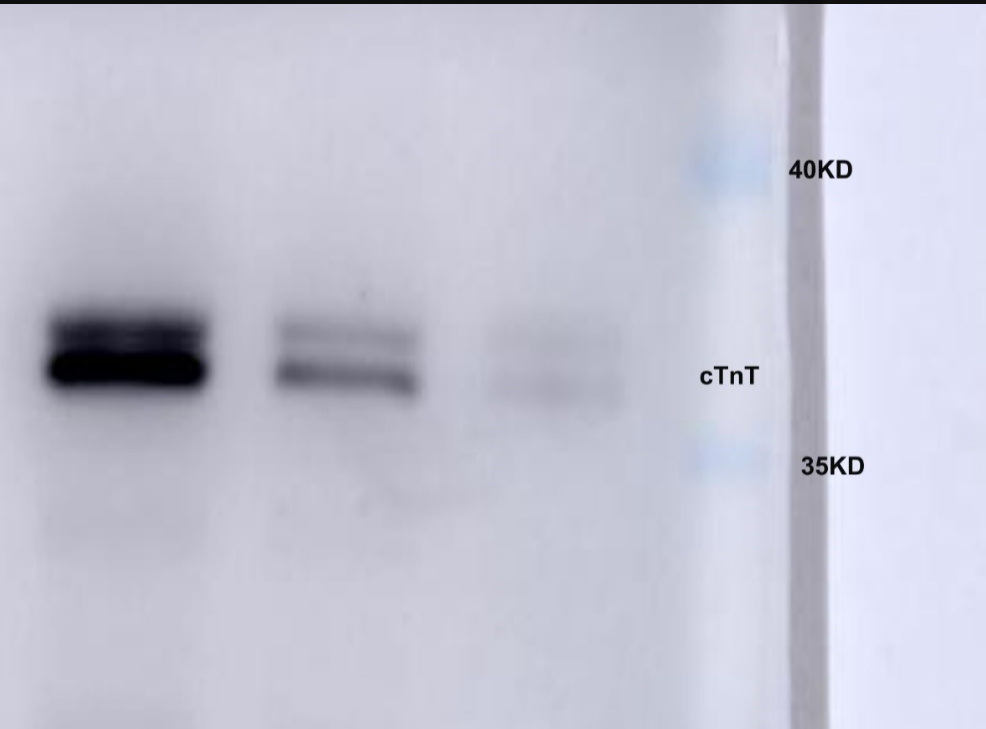

Supplement: Supplementary file 1 — supplementary information [file 41598_2017_15870_MOESM1_ESM.doc]
